# Thrombotic Microangiopathy (TMA) in a Heart Transplant Patient: A Complex Clinical-Pathologic Dilemma

**DOI:** 10.1155/carm/4695420

**Published:** 2025-10-13

**Authors:** Mark A. Colantonio, Bailey Hixon, Kevin Felpel, Md Shahrier Amin, Tahreem Ahmad

**Affiliations:** ^1^Department of Medicine, West Virginia University, Morgantown, West Virginia, USA; ^2^Department of Cardiology, West Virginia University, Morgantown, West Virginia, USA; ^3^Department of Pathology, West Virginia University, Morgantown, West Virginia, USA

**Keywords:** microangiopathic hemolytic anemia, segmental sclerosis, tacrolimus, thrombotic microangiopathy

## Abstract

Drug-induced thrombotic microangiopathy is a rare cause of microangiopathic hemolytic anemia. Tacrolimus is a commonly used immunosuppressive agent postorgan transplant. Due to life-threatening complications associated with microangiopathic hemolytic anemia, it is essential to have a high clinical suspicion for this drug-induced microangiopathy. Here, we present a case of suspected drug-induced thrombotic microangiopathy secondary to long-term tacrolimus use. Although it is difficult to entirely exclude alternative causes of TMA when presented with this rare pathology, clinicians must remain vigilant of this association with tacrolimus use when there is a high suspicion for TMA. To date, few case reports have highlighted this association with chronic calcineurin use. Our case highlights the need for further research into this association.

## 1. Introduction

Thrombotic microangiopathy (TMA) is characterized by the presence of microangiopathic hemolytic anemia (MAHA), thrombocytopenia, and end-organ injury mediated by endothelial damage along with arterial and capillary microthrombi [1–3]. TMA can be further classified as primary or secondary [4]. Primary TMA includes thrombotic thrombocytopenic purpura (TTP) and atypical hemolytic uremic syndrome (aHUS) [4]. Common causes of secondary TMA include pregnancy, infection, medication, and malignancy, among many others [4]. With a high clinical suspicion, appropriate steps must be taken for a prompt and accurate diagnosis, as TMA can lead to life-threatening complications [5]. Hemolytic anemias can present with various findings on peripheral blood smear; however, in TMA, schistocytes or helmet cells are the most common findings [5]. Other findings consistent with MAHA include elevated lactate dehydrogenase (LDH), indirect bilirubin, a negative direct Coombs test, and low haptoglobin [6].

MAHA is a distinct form of hemolytic anemia characterized by nonimmune-mediated destruction of RBCs [5]. This intravascular hemolysis is caused by damage to erythrocytes as they pass through small blood vessels, leading to the fragmentation of free-flowing RBCs and the formation of dense microthrombi that are prone to fragmentation [6]. MAHA may present in a variety of conditions, including pregnancy-related hemolysis (HELLP), infectious etiologies, including hemolytic uremic syndrome (HUS) secondary to Shiga toxin, and a variety of medications [7]. Tacrolimus (FK506), a widely used calcineurin inhibitor (CNI), mediates its effects by binding to the cytosolic protein FKBP12 [8]. Through this interaction, tacrolimus prevents the dephosphorylation and translocation of transcription factors, thereby downregulating the production of inflammatory cytokines [8]. Tacrolimus is commonly used as an immunosuppressive agent in organ transplants, including lung, heart, kidney, and liver, to prevent host-mediated destruction [9]. Although limited case reports have described tacrolimus-induced MAHA [9], it is often difficult to prove the association due to a lack of specific diagnostic laboratory tests and the presence of confounding factors. Therefore, it is important to have a high degree of clinical suspicion and further characterize this potentially life-threatening association in cases with minimal confounding factors. Here, we present a case of tacrolimus-induced MAHA in a heart transplant recipient.

## 2. Case Presentation

The patient is a 71-year-old female with a past medical history significant for hyperlipidemia, hypertension, hypothyroidism, squamous cell carcinoma of the lung status-post lobectomy in 2022, normocytic anemia, anxiety, and ischemic cardiomyopathy secondary to coronary artery disease. She received a heart transplant in 2016 and has been on tacrolimus therapy for immunosuppression since. Recently, she presented to the emergency department (ED) due to symptoms of lightheadedness, dizziness, blood tinged loose stools, and anemia with a hemoglobin level of 5.8 g/dL (11.5–16 g/dL) discovered at her primary care physician's office. In the ED, the patient's creatinine was elevated to 3.5 mg/dL (0.60–1.05 mg/dL) with an estimated glomerular filtration rate (eGFR) of 14 mL/min/BSA (> 60 mL/min/BSA). She was given one unit of packed red blood cells due to her severe anemia and admitted to the medicine service for further workup. Iron studies revealed an iron level of 78 μg/dL (45–170 μg/dL), an iron binding capacity of 281 μg/dL (224–476 μg/dL), an iron saturation of 28% (20%–50%), and a transferrin level of 201 mg/dL (160–340 mg/dL). Serum LDH was elevated to 679 U/L (125–220 U/L), and haptoglobin was decreased < 3 mg/dL (32–197 mg/dL). Folate levels were within normal limits, and vitamin B12 levels were elevated to 1971 pg/mL (200–900 pg/mL). Prothrombin time (PT) was normal. A stool sample was ordered and positive for enteropathogenic *E. coli* (EPEC). Infectious disease was consulted, and the patient was prescribed a 7-day course of ceftriaxone. Peripheral blood smear revealed absolute lymphopenia without circulating blasts and normocytic, normochromic anemia with moderate anisopoikilocytosis. Moderate schistocytes, 3–6 per high-power field, and thrombocytopenia were also observed. Hematology oncology was consulted for further workup.

**Figure 1:**
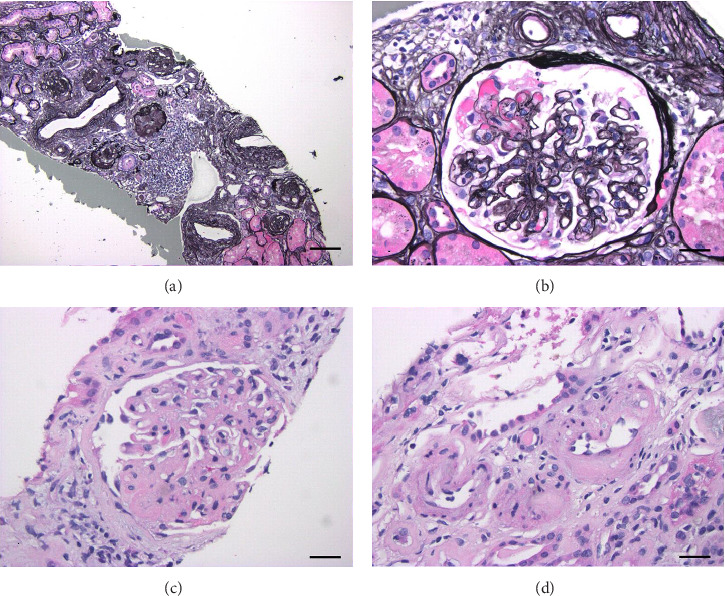
(a) Marked global glomerulosclerosis. (b) Micronangiopathic changes and double contours. (c) Segmental sclerosis in a glomerulus. (d) Arteriolar hyalinosis. (a) and (b) are Jones methenamine silver-stained sections. (c) and (d) are hematoxylin and eosin-stained sections. Scale bar in (a) = 15 μ. Scale barsin (b–d) = 4 μ.

Given the clinical presentation of acute kidney injury, hematochezia, and the presence of pancytopenia, HUS, TTP, disseminated intravascular coagulation (DIC), and tacrolimus-induced MAHA were considered among the differential diagnoses. Due to a concern for tacrolimus-induced TMA, tacrolimus was held. The patient had stabilization of her renal function after discontinuation of tacrolimus, so treatment with other therapies like Eculizumab was not initiated. A kidney biopsy was performed for evaluation of nephritic syndrome and revealed advanced (80%) global glomerulosclerosis, in the setting of microangiopathic vascular changes and sclerosing glomerulonephritis (Figure 1). Her renal biopsy revealed evidence of long-term calcineurin inhibitor use, including marked global and segmental sclerosis and arteriolar hyalinosis. The few remaining glomeruli showed TMA. A definitive etiology for these microangiopathic changes is elusive due to the coexistence of a recent *E. coli* infection and ADAMTS13 deficiency at the time of biopsy. Immunofluorescence studies were negative, suggesting the absence of an immune complex-mediated process. A follow-up peripheral smear revealed similar findings, including mild to moderate schistocytes with 3–5 per high-power field and associated thrombocytopenia. A direct Coombs test was negative, and ADAMTS13 testing was performed, revealing a deficiency of 64% (normal activity > 68%). According to hematology–oncology, it was recommended to discontinue tacrolimus use and transition to an alternative immunosuppressant.

She was transitioned from tacrolimus to sirolimus and continued on mycophenolate. At discharge, the patient's hematochezia resolved, and she was discharged on sirolimus 4 mg per day, mycophenolate 500 mg twice daily, and advised for renal replacement therapy leading to stabilization of her renal function. Unfortunately, the patient had multiple readmissions for recurrent falls and severe deconditioning, and she ultimately expired without renal recovery and clinical improvement.

## 3. Discussion

MAHA is a form of hemolytic anemia secondary to the premature destruction of red blood cells [5]. To date, various etiologies of MAHA have been identified, including DIC, TTP, HUS, HELLP syndrome, and medication-related [7]. In our case, TTP was unable to be entirely ruled out; however, the patient had a Platelets, Lysis, Active cancer, Stem cell or solid organ transplant, MCV, INR, and Creatinine (PLASMIC) score of 4 points, placing her at low risk for TTP. Because of this, TTP was considered less likely. Alternative causes of MAHA, such as DIC, were less likely in the setting of normal PT and PTT; as in typical TMA, these coagulation factors are commonly normal [10]. Here, we describe a case of a patient prescribed long-term tacrolimus therapy post-heart transplant. Although typically associated with Shiga-toxic producing *E. coli*, such as enterohemorrhagic *E. coli*, cases leading to HUS and TMA from EPEC have been described [11]. However, the possibility of a smoldering microangiopathy due to chronic tacrolimus use, leading to global glomerulosclerosis, is not unlikely.

Prior studies have found that roughly 10% of thrombotic microangiopathies are drug-induced [1]. Drug-induced thrombotic microangiopathy (DITMA) exhibits a range of laboratory findings, including elevated LDH, decreased haptoglobin, and the presence of schistocytes in peripheral blood smears [1]. In our case, a peripheral smear revealed mild to moderate schistocytes, with 3–5 per microscopic field, elevated LDH, and deficient haptoglobin, consistent with typical laboratory findings [1]. These findings were compared to the peripheral smear from previous hospitalizations, which was without evidence of schistocytes. Although present in our case, each case may not present with these findings, and clinicians must maintain a high clinical suspicion. Brocklebank et al. found that most patients with progression to MAHA present with acute renal injury with or without thrombocytopenia and hemolytic anemia [12].

As hemolytic anemia can be a life-threatening condition, once suspected, prompt diagnosis is imperative. One should have a high suspicion of drug-induced MAHA with evidence of thrombocytopenia, worsening kidney function, and evidence of schistocytes on peripheral blood smear, all of which our patient had [1]. Furthermore, a decrease in the metalloproteinase ADAMTS13, as observed in our patient, supports the diagnosis [1]. If there remains suspicion for DITMA without a definitive diagnosis, a biopsy can be performed to further aid in diagnosing DITMA. Typical biopsy findings include platelet thrombi with associated fibrosis and microangiopathic glomerular changes, including microthrombi, endothelial swelling, and double contours [12]. Our renal biopsy results showed evidence of long-term CNI use (hyalinosis, global, and segmental sclerosis) and ongoing microangiopathic changes, including microthrombi, as shown in Figure 1. Randhawa et al. described tacrolimus-induced renal injury as epithelial vacuolization and global and segmental sclerosis [13]. Although we noted vacuolization in a rare tubule, this was not a widespread feature in the biopsy to suggest acute CNI toxicity, consistent with the finding of subtherapeutic levels of tacrolimus at presentation.

It is often difficult to pinpoint a single etiologic factor in many cases of TMA. Our case highlights one such clinical scenario. The patient also had two potential etiologies—recent *E. coli* diarrhea and chronic tacrolimus use. However, once suspected, it is most prudent to discontinue and/or replace an offending agent to prevent ongoing damage [9]. In cases where discontinuation of the offending agent is not effective, eculizumab can be used to inhibit progression to renal failure [14] in those at risk. A more cost-effective option, N-acetylcysteine, can also be considered in those without improvement with drug discontinuation [15].

TMA is a life-threatening condition, and prompt identification is essential. Although commonly thought of as an immune reaction, hemolytic anemia may be secondary to medication exposure, leading to the development of microangiopathy and subsequent organ failure. Typical treatment includes drug discontinuation. To date, there have been few extensive, multicenter studies examining the association between medication classes and the development of hemolytic anemia. Our case highlights a common, complicated clinical scenario where patients are on chronic calcineurin use with associated renal injury, and drug-induced MAHA remains a concern. When presented with this rare and complex pathology, one is often unable to entirely exclude other contributing causes to the pathogenesis of TMA, as was the case in our study. Future studies should continue to explore scoring systems to further elucidate the probability and likelihood of specific etiologies of TMA. Ongoing reports and studies describing these complex cases are an essential reminder for clinicians to maintain a broad differential when presented with evidence of typical anemia.

## Data Availability

The data that support the findings of this study are available from the corresponding author upon request.
